# Supplementary material for: A Nationwide Digital Multidisciplinary Intervention Aimed at Promoting Pneumococcal Vaccination in Immunocompromised Patients
Source: Vaccines (Basel). 2023 Aug 11;11(8):1355. doi: 10.3390/vaccines11081355 (PMC10458143; doi:10.3390/vaccines11081355)
Supplement: Supplementary file 1 [file vaccines-11-01355-s001.zip › vaccines-2536465-supplementary.pdf]

Table S1. Characteristics of the targeted intervention population for PPVS23 administration

Variable	Received PPVS23 vaccine during the intervention	Not received PPVS23 vaccine during the intervention	
Age (years) – mean (SD)	54.9 (14.7)	47.7 (13.7)	<0.001
Gender, Female – no. (%)	2904 (50.1)	7757 (55.5)	<0.001
SES			
Low	477 (8.2)	1394 (10)	
Med	3519 (60.8)	8077 (57.8)	<0.001
High	1795 (31)	4495 (32.2)	
Residential community			
General	5256 (90.7)	12,308 (88)	
Ultraorthodox Jews	253 (4.4)	829 (5.9)	<0.001
Arab	286 (4.9)	848 (6.1)	
Current Smoker – no. (%)	822 (14.2)	2186 (15.6)	0.01
Comorbidities – no. (%)			
HTN	2333 (40.3)	3193 (22.8)	<0.001
DM	1201 (20.7)	1551 (11.1)	<0.001
IHD	703 (12.1)	776 (5.5)	<0.001
COPD	261 (4.5)	249 (1.8)	<0.001
Dementia	58 (1)	63 (0.5)	<0.001
Cancer	734 (12.7)	2962 (21.2)	<0.001
OP	1117 (19.3)	1377 (9.8)	<0.001
IBD	949 (16.4)	2167 (15.5)	0.122
Time from entry to registry, (Yr) – mean (SD)	6.0 (5.5)	5.3 (5.2)	<0.001
Number of primary care visits, per month, mean (SD)	1.5 (0.8)	1.1 (0.8)	<0.001
Family physician specialist / intern no. (%)	2614 (45.1)	6137 (43.9)	0.114
Internal / general medicine specialist no. (%)	3181 (54.9)	7848 (56.1)	
Primary care physician age, mean (SD)	54.9 (10.4)	54.5 (10.3)	0.003
